# Renoprotective effects of sodium glucose cotransporter 2 inhibitors in type 2 diabetes patients with decompensated heart failure

**DOI:** 10.1186/s12872-021-02163-7

**Published:** 2021-07-21

**Authors:** Masaki Nakagaito, Teruhiko Imamura, Shuji Joho, Ryuichi Ushijima, Makiko Nakamura, Koichiro Kinugawa

**Affiliations:** 1 Department of Cardiology, Toyama Rosai Hosipital, Toyama, Japan; 2 Second Department of Internal Medicine, University of Toyama, 2630 Sugitani, Toyama, Toyama 930-0194 Japan

**Keywords:** Hemodynamics, Congestion, Chronic kidney disease

## Abstract

**Background:**

Sodium-glucose cotransporter 2 inhibitor (SGLT2i) reduces the risk of the composite renal endpoint and weakens the progressive decline in renal function in patients with chronic heart failure (HF). However, a detailed mechanism of SGLT2i on renal function and outcome remains uninvestigated.

**Methods:**

We prospectively included 40 type 2 diabetic mellitus (T2DM) patients (median 68 years old, 29 male) who were hospitalized for decompensated HF and received SGLT2i during the index hospitalization. Of them, 24 patients had increases in estimated glomerular filtration rate (eGFR) at 12-month follow-up and 16 had decreases in eGFR. We investigated the baseline factors associating with the improvement in renal function.

**Results:**

Lower plasma B-type natriuretic peptide (BNP) level and the use of renin-angiotensin system inhibitor (RASI) were independently associated with increases in eGFR during the follow-up period (*p* < 0.05 for both). Patients with both low plasma BNP levels and uses of RASI achieved significant increases in eGFR irrespective of the baseline HbA1c levels.

**Conclusions:**

Lower plasma BNP level and the use of RASI at baseline were the key factors contributing to the renoprotective effects of SGLT2i among patients with decompensated HF and T2DM.

**Supplementary Information:**

The online version contains supplementary material available at 10.1186/s12872-021-02163-7.

## Background

Patients with heart failure (HF) have a high risk of mortality and morbidity, particularly when they have concomitant kidney impairment [1, 2]. Impaired renal function is common in patients with HF and reduced ejection fraction (HFrEF) and up to 50% of them have chronic kidney disease (CKD) [3]. Patients with CKD also commonly develop HF, and their dominant cause of death is a cardiovascular event.

The currently approved medication to protect kidney function in patients with type 2 diabetes mellitus (T2DM) is renin-angiotensin system inhibitor (RASI) [4, 5]. Sodium-glucose cotransporter 2 inhibitor (SGLT2i), which ameliorates hyperglycemia by suppressing renal glucose reabsorption in urine, has been demonstrated to have favorable effects on the kidney and cardiovascular outcomes in large clinical trials involving patients with T2DM [6–8]. The EMPEROR-Reduced trial further demonstrated that SGLT2i was associated with a lower risk of composite renal outcome and a slower progressive decline in renal function in patients with HFrEF, irrespective of the existence of T2DM [9]. These studies suggest that the renal benefit of SGLT2i appears to be independent of their blood glucose-lowering effects.

However, its detailed mechanism remains uninvestigated. Detailed assessments of the renoprotective effect of SGLT2i would be a key to more clarify the clinical implication and optimal patient selection to further enjoy the renoprotective effect of the SGLT2i. As a preliminary step, we investigated the factors associating with the improvement in renal function (super-response in renoprotective effect) during the SGLT2i therapy in patients with HF and T2DM.

## Methods

The present study was a single-center, non-randomized, open-labeled, prospective registry study designed to assess the factors associating with the renoprotection of SGLT2i therapy for the patients with HF and T2DM. The local Institutional Ethics Board approved the study protocol (#Rin 29-94), which complied with the Declaration of Helsinki. Written informed consent was obtained from all of the patients beforehand.

### Study population

This study involved consecutive T2DM patients who had received SGLT2i for the first time during their index hospitalization for decompensated HF, which was diagnosed according to the Framingham criteria, at our institute between February 2016 and September 2019. All patients had New York Heart Association (NYHA) class III/IV symptoms upon admission. Among canagliflozin (100 mg/day), dapagliflozin (5 mg/day), and empagliflozin (10 mg/day), one SGLT2i was non-randomly selected and administered. All patients had HbA1c level of 6.1% or higher and received guideline-directed medical therapy for HF.

Exclusion criteria were as follows: type 1 diabetes mellitus, end-stage renal failure (estimated glomerular filtration rate (eGFR) < 20 mL/min/1.73m^2^), use of any mechanical circulatory supports, pregnancy or breastfeeding in the study period, history of hypersensitivity to the study drugs, severe ketosis, diabetic coma or precoma, and suspension of SGLT2i during the observation period.

### Study design and data collection

Baseline characteristics including demographics and laboratory data were obtained at index discharge. eGFR at baseline and 6 and 12 months after discharge was retrospectively retrieved. A primary endpoint was defined as any increases in eGFR at 12 months after discharge compared with the index discharge (“super-response” in the achievement of renoprotection). The eGFR was calculated using the guidelines from the Chronic Kidney Disease Epidemiology Collaboration.

### Statistical analyses

Continuous variables were expressed as the median and interquartile unless any specific statements. Categorical variables were expressed as absolute numbers and percentages. Wilcoxon test was applied to compare continuous parameters, and Pearson’s χ^2^ test was applied for comparison of categorical variables. Trends of continuous variables were compared using the Friedman test. Univariable and multivariable analyses with logistic regression models were performed to calculate the adjusted odds ratio to assess the influence of various parameters on the renoprotective effect of SGLT2i. Variables significant with *p* < 0.05 in the univariate analyses were included in the multivariate analyses. A cut-off of plasm B-type natriuretic peptide (BNP) concentration for any increases in eGFR after SGLT2i initiation was calculated using receiver operating characteristic analysis. The statistical analysis was performed by using JMP® 15 (SAS Institute Inc., Cary, NC, USA). The level of significance was defined as *p* < 0.05.

## Results

### Baseline characteristics

**Fig. 1:**
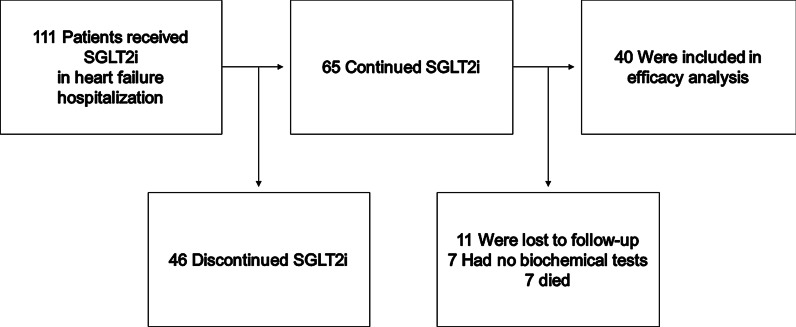
Enrollment and follow-up

A total of 111 patients were considered to be included (Fig. 1). Of them, 65 patients continued SGLT2i without suspension for 12 months. 18 patients who were lost to follow-up or had no biochemical tests were excluded. Cardiovascular death occurred in 4 patients and non-cardiovascular death occurred in 3 patients. A total of 40 patients (median 68 years old, 29 male) were finally included in this study.

The baseline characteristics are summarized in Table 1. Median age was 68 years old and 29 were male. Baseline eGFR was 53.0 (36.1, 74.5) mL/min/1.73m^2^.

**Table 1:** Baseline characteristics

	Total (N = 40)	Increased eGFR (N = 26)	No-increased eGFR (N = 14)	P value
Age, years	68 (57–75)	68 (55–72)	71 (61–79)	0.177
Male, N	29 (73)	19 (73)	10 (71)	0.911
Body weight, kg	62 (51–73)	66 (54–76)	54 (47–71)	0.112
Body mass index, kg/m^2^	23.9 (19.6–27.2)	24.7 (21.9–27.9)	20.4 (19.0–24.9)	0.076
Systolic blood pressure, mmHg	108 (95–119)	110 (100–119)	100 (90–120)	0.173
Heart rate, beats per minutes	70 (63–81)	69 (63–83)	74 (64–79)	0.570
HbA1c, %	6.8 (6.6–7.6)	6.7 (6.6–7.7)	7.0 (6.6–7.2)	0.776
Fasting blood sugar, mg/dL	110 (96–129)	102 (86–128)	122 (107–130)	0.076
Left ventricular ejection fraction, %	42 (27–56)	39 (28–58)	42 (26–55)	0.966
Ischemic etiology, N	19 (48)	12 (46)	7 (50)	0.816
Atrial fibrillation, N	8 (20)	5 (19)	3 (21)	0.868
Hemoglobin, g/dL	12.9 (11.5–15.6)	13.6 (16.0–11.7)	12.4 (11.1–14.2)	0.223
Hematocrit, %	38.5 (34.2–44.9)	40.6 (35.2–45.6)	37.6 (33.5–41.4)	0.192
Serum albumin, g/dL	3.7 (3.5–3.8)	3.7 (3.5–3.9)	3.6 (3.2–3.7)	0.107
Serum sodium, mEq/L	138 (136.3–140)	139.5 (137.8–140)	137 (134.5–140.3)	0.094
Serum potassium, mEq/L	4.4 (4.1–4.6)	4.4 (4.1–4.6)	4.4 (4.2–4.5)	0.943
eGFR, mL/minute/1.73m^2^	53.0 (36.1–74.5)	54.5 (40.0–73.5)	50.1 (31.6–80.4)	0.712
Plasma BNP, pg/mL	94 (54–251)	86 (50–175)	205 (75–374)	0.059
Plasma NT-proBNP, pg/mL	864 (260–1819)	740 (251–1424)	1479 (356–3057)	0.242
*Heart failure therapies*				
Beta-blockers, N	37 (93)	23 (89)	14 (100)	0.186
ACEI/ARB, N	37 (93)	26 (100)	11 (79)	0.014
Loop diuretics, N	19 (48)	11 (42)	8 (57)	0.370
Furosemide, mg/day	0 (0–20)	0 (0–20)	20 (0–40)	0.158
MRA, N	27 (68)	17 (65)	10 (71)	0.697
Thiazides, N	2 (5)	2 (8)	0 (0)	0.287
Statin, N	30 (75)	20 (77)	10 (71)	0.702
*Anti-diabetic agents*				
Sulfonylureas, N	3 (8)	2 (8)	1 (7)	0.950
DPP-4i, N	20 (50)	14 (54)	6 (43)	0.741
Biguanides, N	8 (20)	5 (19)	3 (21)	0.868
Insulin, N	5 (12)	5 (19)	0 (0)	0.079

Continuous variables were expressed as median (25%-75% percentile) and categorical variables were expressed as number (%)

HbA1c, glycated hemoglobin; eGFR, estimated glomerular filtration rate; BNP, b-type natriuretic peptide; NT-proBNP, N-terminal pro-b-type natriuretic peptide; ACEI, angiotensin converting enzyme inhibitors; ARB, angiotensin receptor blockers; MRA, mineralocorticoid receptor antagonists; DPP-4i, dipeptidyl peptidase-4 inhibitors

### Discontinued group

Among 46 patients who discontinued SGLT2i during the index hospitalization, 19 were followed for 12 months. There were no statistically significant differences in the baseline characteristics except for age and the dose of furosemide (Additional file 1: Table 1).

There were no significant differences in the eGFR at baseline between both groups. By contrast, eGFR tended to be higher in continued group compared to discontinued group 6 and 12 months after discharge (Additional file 2: Figure 1). Only age and continuation of SGLT2i were significantly correlated with the changes in eGFR during the 12-month follow-up period (Additional file 3: Table 2).

### Achievement of the primary endpoint

**Fig. 2:**
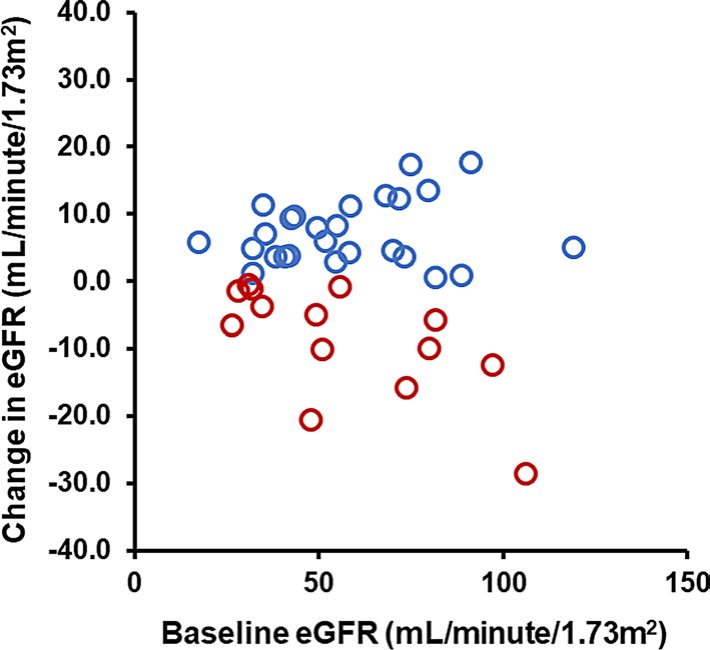
Distribution of the changes in estimated glomerular filtration rate (eGFR)

The participants were divided into two groups according to the achievement of primary endpoint: any increases in eGFR at 12-month follow-up: an increased group (N = 26) and a decreased group (N = 14). The median value of changing eGFR (eGFR at month 12—eGFR at baseline) in the two groups was + 5.5 and − 7.5, respectively (Fig. 2). *25/40 had anti-diabetic agents and 20/40 had* history of hospitalization for HF at the time of index hospitalization. No patients initiated anti-heart failure medications during the observational period.

### Baseline characteristics stratified by the achievement of primary endpoint

There were no significant differences in most of the demographic data between the two groups. The prescription rate of RASI was higher in the increased eGFR group. The proportions of participants taking oral hypoglycemic agents were statistically not different between the two groups. Baseline plasma BNP level tended to be lower in the increased eGFR group. Of note, the baseline eGFR value was not statistically different between the two groups.

### Variables associating with renoprotection during SGLT2i therapy

In univariate logistic regression analysis, body mass index, plasma BNP level, usage of RASI, and insulin administration were significantly associated with the renoprotective effect of SGLT2i (*p* < 0.05 for all; Table 2). Lower plasma BNP level (0.26 of odds ratio, 95% confidence interval 0.08–0.79) and the use of RASI were independently associated with the renoprotective effect of SGLT2i (*p* < 0.05 for both). Of note, no patients achieved the primary endpoints without RASI. This finding was not confirmed in the logistic regression analysis with both patients with and without SGLT2i continuation (Additional file 4: Table 3).

**Table 2:** Logistic regression analyses for any increases in eGFR

Variables	All patients (N = 40)					
	Univariate analysis			Multivariate analysis		
	95% CI	Odds ratio	*p* value	95% CI	Odds ratio	*p* value
Age	–	–	0.154			
Male	–	–	0.912			
Body mass index	0.99–1.39	1.17	0.041	–	–	0.265
Systolic blood pressure	–	–	0.145			
Heart rate	–	–	0.671			
HbA1c	–	–	0.520			
Fasting blood sugar	–	–	0.311			
Left ventricular ejection fraction	–	–	0.797			
Ischemic etiology	–	–	0.816			
Atrial fibrillation	–	–	0.869			
Hemoglobin	–	–	0.199			
Hematocrit	–	–	0.176			
Serum albumin	–	–	0.068			
Serum sodium	–	–	0.062			
Serum potassium	–	–	0.685			
eGFR	–	–	0.897			
BNP (per 163 pg/mL increase)	0.16–0.88	0.37	0.016	0.08–0.79	0.26	0.007
NT-proBNP	–	–	0.163			
Beta–blockers	–	–	0.100			
ACEI/ARB	NA	NA	0.009	NA	NA	0.002
Loop diuretics	–	–	0.370			
Furosemide (per 10 mg/day increase)	–	–	0.077			
MRA	–	–	0.696			
Thiazides	–	–	0.182			
Statin	–	–	0.704			
Sulfonylureas	–	–	0.950			
DPP-4i	–	–	0.507			
Biguanides	–	–	0.869			
Insulin	NA	NA	0.030	–	–	0.103

HbA1c, glycated hemoglobin; eGFR, estimated glomerular filtration rate; BNP, b-type natriuretic peptide; NT-proBNP, N-terminal pro-b-type natriuretic peptide; ACEI, angiotensin converting enzyme inhibitors; ARB, angiotensin receptor blockers; MRA, mineralocorticoid receptor antagonists; DPP-4i, dipeptidyl peptidase-4 inhibitors

Odds ratio were not calculated in several variables due to statistical divergence

### Stratification of the primary endpoint using BNP and RASI use

**Fig. 3:**
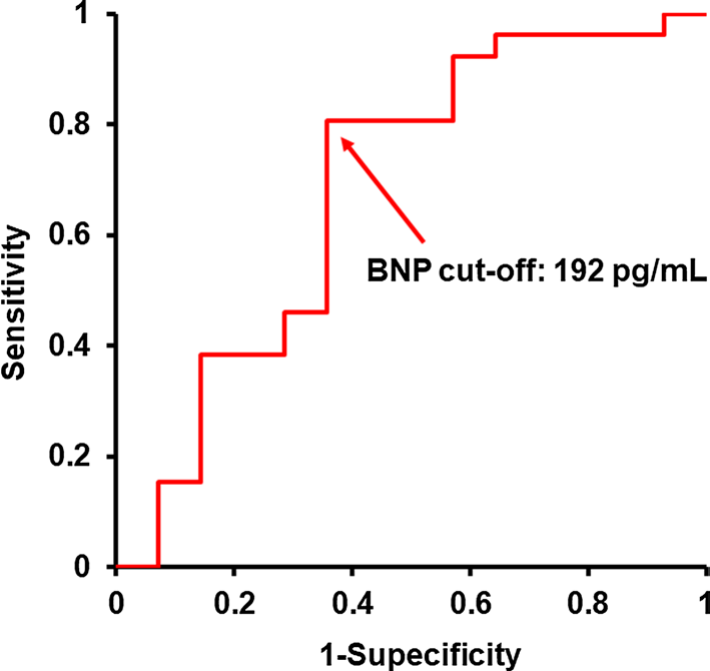
Receiver operating characteristic (ROC) curve of baseline plasma BNP level

A cut-off of baseline plasma BNP level to predict the primary endpoint was 192 pg/mL (0.684 of area under the curve, 0.808 of sensitivity, and 0.643 of specificity; Fig. 3).

**Fig. 4:**
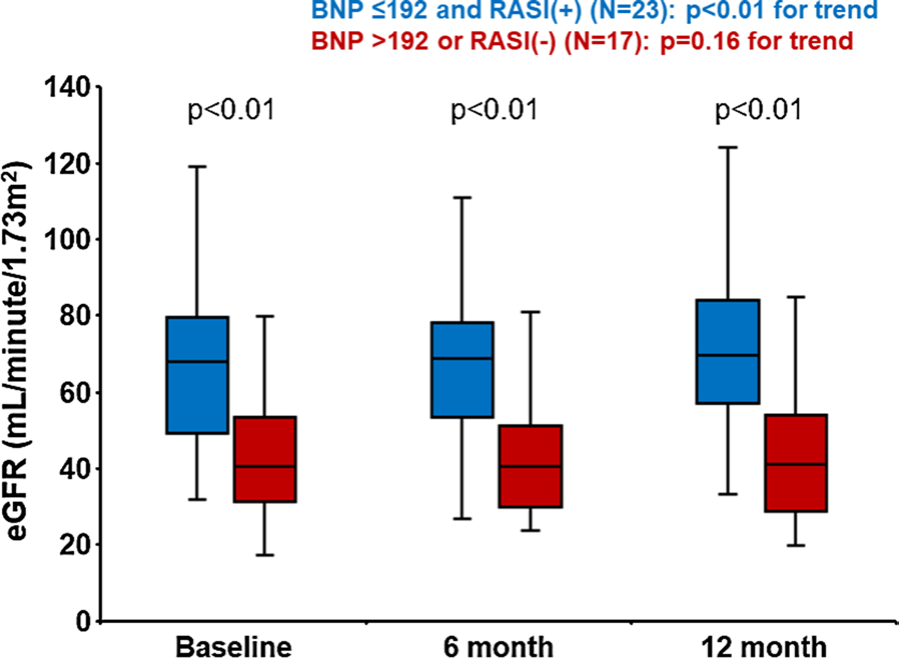
Changes in estimated glomerular filtration rate (eGFR) during the 1-year observational period. Variables were expressed as mean and standard deviations

Twenty-three patients satisfied both plasma BNP < 192 pg/mL and RASI use (double-positive group). eGFR increased significantly during the 12-month follow-up in the double-positive group, whereas eGFR remained unchanged in the no double positive group (Fig. 4).

**Fig. 5:**
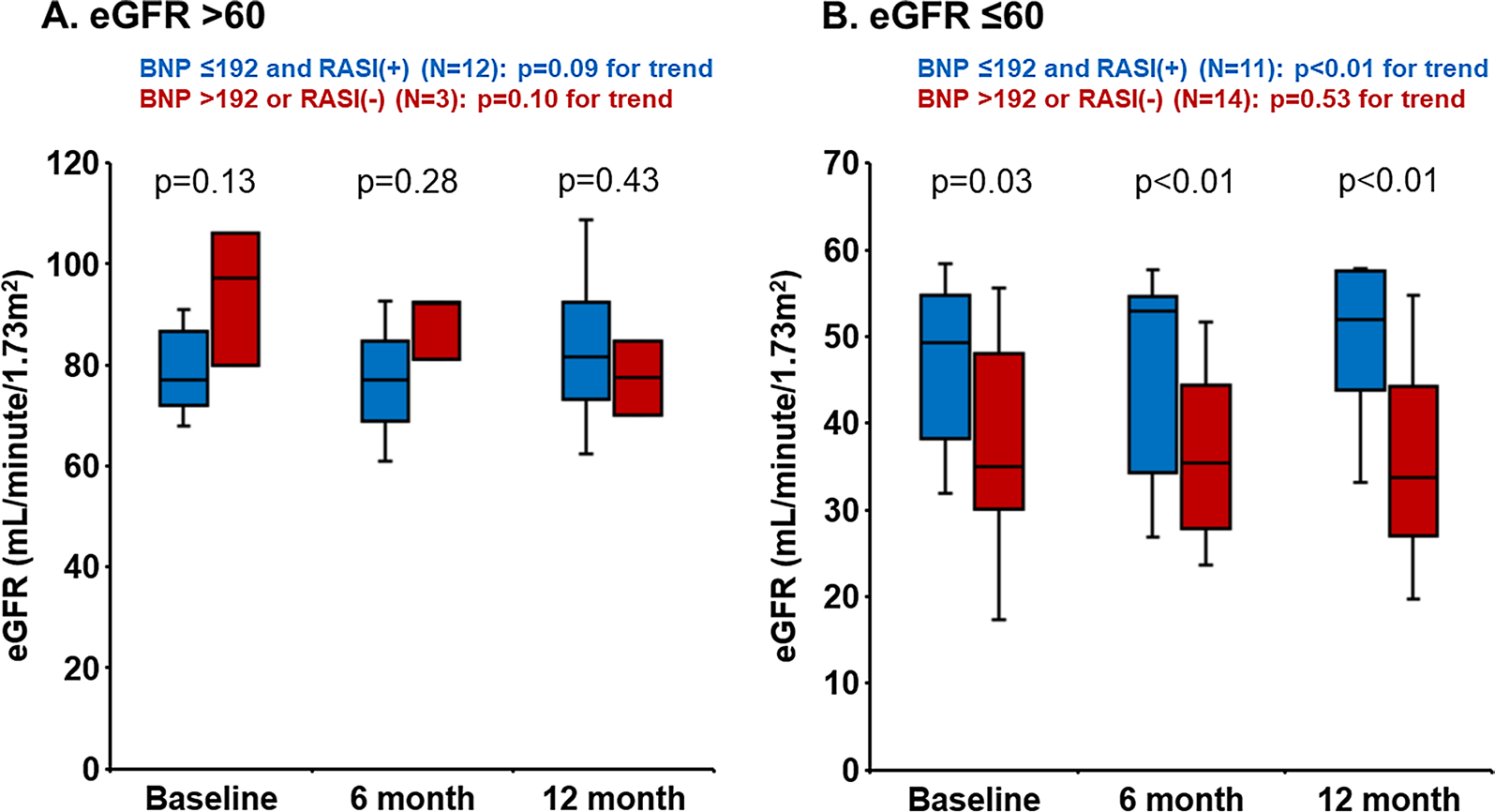
Changes in estimated glomerular filtration rate (eGFR) during the one-year observational period stratified by baseline eGFR. Variables were expressed as mean and standard deviations

**Fig. 6:**
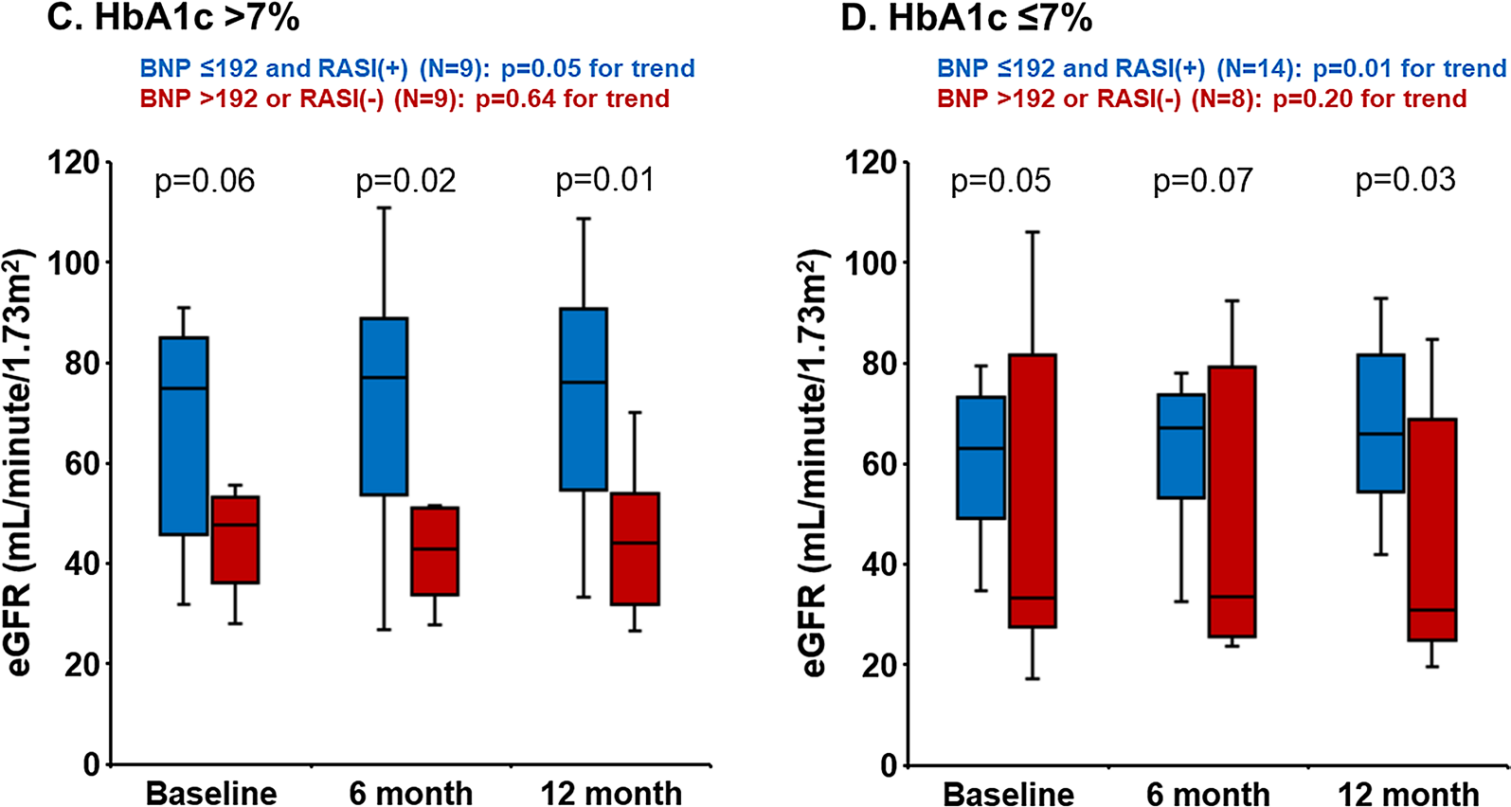
Changes in estimated glomerular filtration rate (eGFR) during the one-year observational period stratified by baseline glycated hemoglobin (HbA1c) levels. Variables were expressed as mean and standard deviations

As sub-analyses, similar trends were observed irrespective of the eGFR levels stratified by 60 mL/min/1.73m^2^ (Fig. 5AB) and the HbA1c levels stratified by 7.0% (Fig. 6CD).

## Discussion

We investigated the factors associating with the renoprotection during SGLT2i therapy in patients with HF and T2DM. The major finding of the present study was that lower plasma BNP level and the use of RASI at baseline were associated with the renoprotective effect of SGLT2i. Those with lower plasma BNP levels and the use of RASI had greater eGFR during the 12-month follow-up period over those with neither of them irrespective of the eGFR levels and the HbA1c levels at baseline).

### SGLT2i and BNP

Both EMPEROR-Reduced trial and DAPA-HF trial demonstrated that SGLT2i prevented the occurrence of worsening HF in patients with HFrEF [9, 10]. The EMPEROR-Reduced trial further demonstrated that empagliflozin was associated with a lower risk of renal outcome and a slower progressive decline in renal function. Several large placebo-controlled trials suggested that SGLT2i might exert a beneficial effect on the renal outcome as a class effect [6–8]. On the contrary, the DAPA-HF trial, which used dapagliflozin, did not demonstrate the improvement of renal outcome [11].

The pattern of inconsistent findings in renal outcomes might be explained by the differences in the distribution of NYHA functional class in each trial. More patients with NYHA class II were enrolled in the EMPEROR-Reduced trial compared to the DAPA-HF trial. SGLT2i might have renoprotective effect particularly for those with less sick HF, as we also found in this study. In a meta-analysis of the EMPEROR-Reduced and DAPA-HF trials, HFrEF patients with NYHA class II also had a lower risk of composite cardiovascular outcome compared to those with NYHA class III–IV symptoms [12].

These findings appear to be due to direct cardioprotective and nephroprotective effects of SGLT2i, which may be related to actions on sodium balance, energy homeostasis, and mitigation of cellular stress [13]. The detailed mechanism remains uncertain, but the existence of renal congestion, indicated by the elevated BNP level, might suppress the improvement in renal function during SGLT2i therapy.

### SGLT2i and RASI

RASI were the only classes of medication that slows a decline in kidney function [4, 5]. The concomitant use of RASI together with SGLT2i was associated with the renoprotective effect also in the present study. Although few studies have investigated the combined effects of RASI and SGLT2i, several previous studies of SGLT2i have identified a minimal increase in plasma renin activity [14, 15]. SGLT2i can cause diuresis, natriuresis, and associated body fluid loss, resulting in renin activation, whereas RASI may counterbalance this effect. Hence, RASI may have played an important role in renal protection in the present study.

Conversely, several studies in animal models or humans have confirmed unchanged activity in the renin–angiotensin–aldosterone system following the SGLT2i administration [16, 17]. An increase in GFR associating with long-term SGLT2i therapy is thought to be secondary to tubuloglomerular feedback, which is also a response of the macula densa to the increased salt delivery via inhibition of sodium transport proximally [18]. Furthermore, an increase in sodium chloride delivery to the macula densa may suppress the renin–angiotensin–aldosterone system. These different effects of SGLT2i may explain the inconsistent data regarding the responses of renin–angiotensin–aldosterone system to SGLT2i.

Consequently, the association between SGLT2i and systemic renin–angiotensin–aldosterone system activation is not straightforward. However, since plasma renin activity is significantly higher in patients with HF compared to healthy people [19], it is plausible that the renin–angiotensin–aldosterone system is activated in participants in the present study. This hypothesis may explain the finding that the use of RASI was associated with the renoprotective effect of SGLT2i in the present study.

### Limitations

The sample size was small and the observation period was only one year. If 0.05 of alpha error, 0.85 of 1-beta, and 0.9 of effect size was set, a total of 48 participants were statistically required. Given the low event number, the number of potential confounders included in the multivariate analyses was restricted. The one-year observation period may be insufficient to assess changes in eGFR after SGLT2i administration. In addition, since we investigated the changes in renal function from discharge rather than after SGLT2i administration, we could not confirm the initial changes in eGFR after starting SGLT2i in this study. A large-scale multicenter study with a longer follow-up period is required.

The multiple types of SGLT2i were used in the present study. It remains unclear whether the renal beneficial effect is consistent across any SGLT2is.

Unlike EMPEROR-Reduced trial and DAPA-HF trial, we did not have any data of proteinuria to assess renal function. Also, we defined renoprotective effect as any increase in eGFR (primary endpoint) to investigate “super-responder” to SGLT2i, whereas patients included in these trials showed gradual decline in eGFR during SGLT2i therapy. Our findings might not simply be applicable to other studies with different definition of renoprotective effect.

Lastly, the EMPEROR-Reduced trial revealed that SGLT2i reduced the risk of the composite renal endpoint, independently of diabetes status [20]. We also indicated that increases in eGFR during the observation period were independent of the HbA1c levels, whereas we did not include patients without T2DM in this study. Further studies are warranted to clarify the mechanism of SGLT2i on renal function and outcome.

## Conclusions

Lower plasma BNP level and the use of RASI at baseline were the possible factors contributing to the improvement in renal function among HF and T2DM patients during the SGLT2i therapy.

## Supplementary Information

**Additional file 1: Table 1.**. Baseline characteristics.

**Additional file 2: Figure 1.** Trends in eGFR between those with and without SGLT2i continuation.

**Additional file 3: Table 2.** Logistic regression analyses for increases in eGFR in both patients who continued and discontinued SGLT2i.

**Additional file 4: Table 3.** Logistic regression analyses for increases in eGFR in both patients who continued and discontinued SGLT2i.

## Data Availability

The datasets used and/or analysed during the current study are available from the corresponding author on reasonable request.

## Abbreviations

HF: Heart failure
HFrEF: Heart failure and reduced ejection fraction
CKD: Chronic kidney disease
T2DM: Type 2 diabetes mellitus
RASI: Renin-angiotensin system inhibitor
SGLT2i: Sodium-glucose cotransporter 2 inhibitor
NYHA: New York Heart Association
eGFR: Estimated glomerular filtration rate
BNP: B-type natriuretic peptide
