# Supplementary material for: Renoprotective effects of sodium glucose cotransporter 2 inhibitors in type 2 diabetes patients with decompensated heart failure
Source: BMC Cardiovasc Disord. 2021 Jul 21;21:347. doi: 10.1186/s12872-021-02163-7 (PMC8296582; doi:10.1186/s12872-021-02163-7)
Supplement: Supplementary file 1 — Additional file 1: Table 1.. Baseline characteristics. [file 12872_2021_2163_MOESM1_ESM.docx]

| **Supplementary Table 1. Baseline characteristics** | | | | |
| --- | --- | --- | --- | --- |
|  | **Total**  **(N = 59)** | **Continuation**  **of SGLT2i**  **(N = 40)** | **Discontinuation**  **Of SLT2i**  **(N = 19)** | **P value** |
| Age, years | 69 (78-61) | 68 (57-75) | 72 (68-82) | 0.032 |
| Male, N | 39 (66) | 29 (73) | 10 (53) | 0.132 |
| Body weight, kg | 58 (49-72) | 62 (51-73) | 54 (48-65) | 0.116 |
| Body mass index, kg/m^2^ | 23.4 (19.8-26.4) | 23.9 (19.6-27.2) | 21.9 (19.9-24.4) | 0.267 |
| Systolic blood pressure, mmHg | 108 (96-118) | 108 (95-119) | 100 (97-113) | 0.460 |
| Heart rate, beats per minutes | 72 (63-84) | 70 (63-81) | 77 (63-89) | 0.266 |
| HbA1c, % | 6.8 (6.5-7.6) | 6.8 (6.6-7.6) | 6.8 (6.4-7.9) | 0.666 |
| Fasting blood sugar, mg/dL | 110 (94-129) | 110 (96-129) | 116 (88-134) | 1.000 |
| Left ventricular ejection fraction, % | 39 (27-54) | 42 (27-56) | 34 (25-50) | 0.359 |
| Ischemic etiology, N | 29 (49) | 19 (48) | 10 (53) | 0.713 |
| Atrial fibrillation, N | 15 (25) | 8 (20) | 7 (37) | 0.165 |
| Hemoglobin, g/dL | 12.3 (11.1-14.9) | 12.9 (11.5-15.6) | 11.9 (9.9-14.5) | 0.036 |
| Hematocrit, % | 37.3 (33.5-43.8) | 38.5 (34.2-44.9) | 35.6 (30.8-42.8) | 0.060 |
| Serum albumin, g/dL | 3.6 (3.4-3.8) | 3.7 (3.5-3.8) | 3.5 (3.0-3.8) | 0.059 |
| Serum sodium, mEq/L | 138 (135-140) | 138 (136-140) | 137 (134-138) | 0.074 |
| Serum potassium, mEq/L | 4.4 (4.1-4.7) | 4.4 (4.1-4.6) | 4.4 (4.0-4.8) | 0.526 |
| eGFR, mL/minute/1.73m^2^ | 51.9 (34.5-73.0) | 53.0 (36.1-74.5) | 51.9 (32.1-62.3) | 0.491 |
| Plasma BNP, pg/mL | 127 (75-261) | 94 (54-251) | 154 (102-302) | 0.118 |
| Plasma NT-proBNP, pg/mL | 1112 (387-1917) | 864 (260-1819) | 1554 (626-2699) | 0.057 |
| Heart failure therapies |  |  |  |  |
| Beta-blockers, N | 53 (90) | 37 (93) | 16 (84) | 0.325 |
| ACEI/ARB, N | 52 (88) | 37 (93) | 15 (79) | 0.133 |
| Loop diuretics, N | 34 (58) | 19 (48) | 15 (79) | 0.022 |
| Furosemide, mg/day | 20 (0-20) | 0 (0-20) | 20 (10-40) | 0.025 |
| MRA, N | 42 (71) | 27 (68) | 15 (79) | 0.364 |
| Thiazides, N | 2 (3) | 2 (5) | 0 (0) | 0.321 |
| Statin, N | 45 (76) | 30 (75) | 15 (79) | 0.739 |
| Anti-diabetic agents |  |  |  |  |
| Sulfonylureas, N | 5 (9) | 3 (8) | 2 (11) | 0.697 |
| DPP-4i, N | 30 (51) | 20 (50) | 10 (53) | 0.850 |
| Biguanides, N | 9 (15) | 8 (20) | 1 (5) | 0.141 |
| Insulin, N | 9 (15) | 5 (12) | 4 (21) | 0.393 |
| HbA1c, glycated hemoglobin; eGFR, estimated glomerular filtration rate; BNP, b-type natriuretic peptide; NT-proBNP, N-terminal pro-b-type natriuretic peptide; ACEI, angiotensin converting enzyme inhibitors; ARB, angiotensin receptor blockers; MRA, mineralocorticoid receptor antagonists; DPP-4i, dipeptidyl peptidase-4 inhibitors  Continuous variables were expressed as median (25%-75% percentile) and categorical variables were expressed as number (%) | | | | |
