# Supplementary figures and images for: Renoprotective effects of sodium glucose cotransporter 2 inhibitors in type 2 diabetes patients with decompensated heart failure
Source: BMC Cardiovasc Disord. 2021 Jul 21;21:347. doi: 10.1186/s12872-021-02163-7 (PMC8296582; doi:10.1186/s12872-021-02163-7)

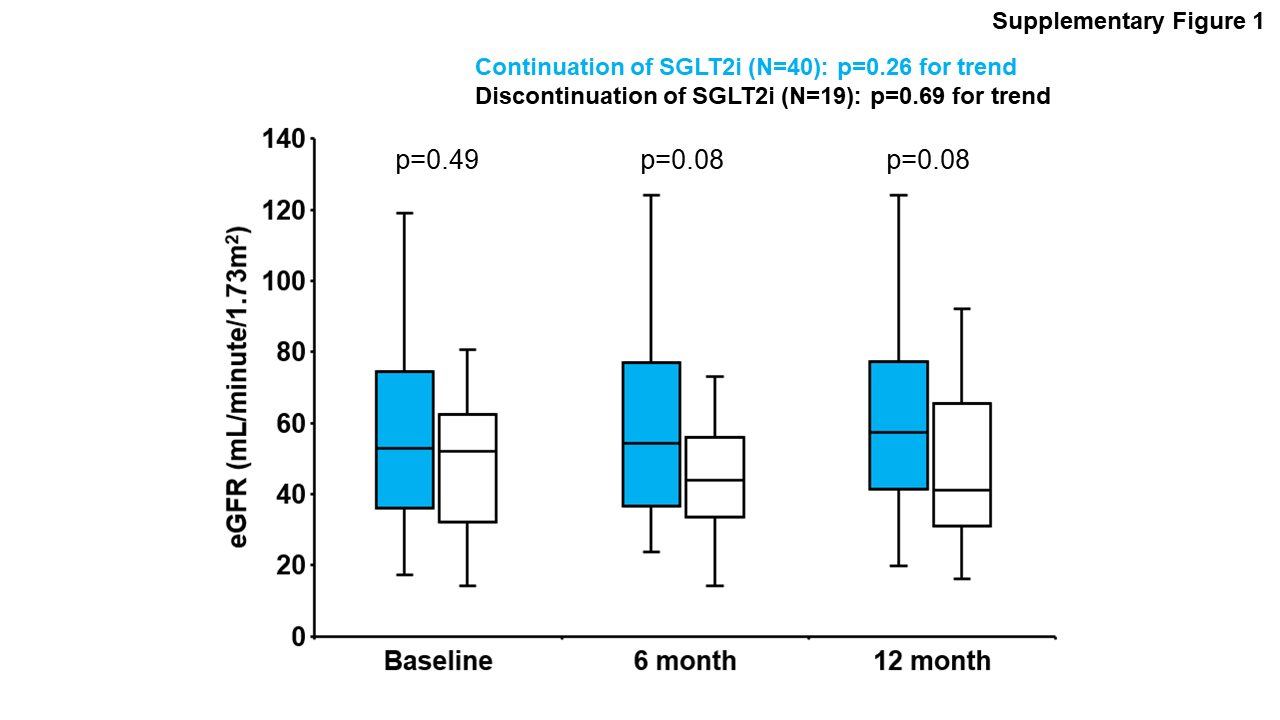

Supplement: Supplementary file 2 — Additional file 2: Figure 1. Trends in eGFR between those with and without SGLT2i continuation. [file 12872_2021_2163_MOESM2_ESM.tif]
