# Supplementary material for: Renoprotective effects of sodium glucose cotransporter 2 inhibitors in type 2 diabetes patients with decompensated heart failure
Source: BMC Cardiovasc Disord. 2021 Jul 21;21:347. doi: 10.1186/s12872-021-02163-7 (PMC8296582; doi:10.1186/s12872-021-02163-7)
Supplement: Supplementary file 4 — Additional file 4: Table 3. Logistic regression analyses for increases in eGFR in both patients who continued and discontinued SGLT2i. [file 12872_2021_2163_MOESM4_ESM.docx]

**Supplementary Table 3. Logistic regression analyses for increases in eGFR in both patients** **who continued and discontinued SGLT2i**

|  | **All patients (N = 59)** | | | | | |
| --- | --- | --- | --- | --- | --- | --- |
|  | **Univariate analysis** | | | **Multivariate analysis** | | |
| **Variables** | **95% CI** | **Odds ratio** | **p value** | **95% CI** | **Odds ratio** | **p value** |
| Age | - | - | 0.398 |  |  |  |
| Male | - | - | 0.161 |  |  |  |
| Body mass index | 0.72-0.97 | 0.84 | 0.010 | 0.72-1.01 | 0.85 | 0.050 |
| Systolic blood pressure | - | - | 0.775 |  |  |  |
| Heart rate | - | - | 0.170 |  |  |  |
| HbA1c | - | - | 0.693 |  |  |  |
| Fasting blood sugar | - | - | 0.460 |  |  |  |
| Left ventricular ejection fraction | - | - | 0.862 |  |  |  |
| Ischemic etiology | - | - | 0.152 |  |  |  |
| Atrial fibrillation | - | - | 0.829 |  |  |  |
| Hemoglobin | - | - | 0.078 |  |  |  |
| Hematocrit | - | - | 0.085 |  |  |  |
| Serum albumin | 0.03-0.65 | 0.14 | 0.006 | 0.03-0.87 | 0.16 | 0.025 |
| Serum sodium | - | - | 0.103 |  |  |  |
| Serum potassium | - | - | 0.215 |  |  |  |
| eGFR | - | - | 0.510 |  |  |  |
| BNP (per 174 pg/mL increase) | 1.29-6.20 | 2.83 | 0.005 | - | - | 0.123 |
| NT-proBNP | - | - | 0.160 |  |  |  |
| Beta-blockers | - | - | 0.157 |  |  |  |
| ACEI/ARB | - | - | 0.098 |  |  |  |
| Loop diuretics | - | - | 0.394 |  |  |  |
| Furosemide (per 10 mg/day increase) | - | - | 0.466 |  |  |  |
| MRA | - | - | 0.906 |  |  |  |
| Thiazides |  |  | 0.133 |  |  |  |
| Statin |  |  | 0.967 |  |  |  |
| Sulfonylureas | - | - | 0.270 |  |  |  |
| DPP-4i | - | - | 0.708 |  |  |  |
| Biguanides | - | - | 0.892 |  |  |  |
| Insulin | - | - | 0.892 |  |  |  |
| HbA1c, glycated hemoglobin; eGFR, estimated glomerular filtration rate; BNP, b-type natriuretic peptide; NT-proBNP, N-terminal pro-b-type natriuretic peptide; ACEI, angiotensin converting enzyme inhibitors; ARB, angiotensin receptor blockers; MRA, mineralocorticoid receptor antagonists; DPP-4i, dipeptidyl peptidase-4 inhibitors.  Odds ratio were not calculated in several variables due to statistical divergence. | | | | | | |
